# The Book World of Medicine and Science

**Published:** 1907-01-05

**Authors:** 


### THE BOOK WORLD OF MEDICINE AND SCIENCE.

THE OTHER SIDE OF THE LANTERN: AN ACCOUNT OF A COMMONPLACE TOUR ROUND THE WORLD. By Sir FREDERICK TREVES, Bart., G.C.V.O., C.B., LL.D., Sergeant-Surgeon to H.M. the King, Surgeon-in-Ordinary to H.R.H. the Prince of Wales, Author of "The Tales of a Field Hospital." With forty illustrations from photographs. Popular edition. (London: Cassell and Company. 6s.)

THE tour is not commonplace. There are few books of travel in which scenes are described and figures depicted with such startling vividness as in this volume. Travellers' tales are sometimes tedious, frequently even ugly and indistinct. In this work the passion, opinion, and ambition of our fellow-men in different parts of the globe are interspersed with descriptions of interesting facts delightfully portrayed. It is not everybody who has had the happy chance of finding material for such a work, and it is fortunate that fate has so favoured the readers that such an opportunity should have been taken advantage of by Sir Frederick Treves. Fluency of style, sound reasoning, easy wit, cynical comparisons, full material, and a blending of the whole with the tact of the biographer and the eye of the artist, bespeak the writer with all his individuality of character and human interests. Now and again he gives us a touch of the spirit of cynicism, almost Scottish in its causticity. His comparisons are characteristic, as, for instance, when he compares an ironclad in the Bay of Gibraltar to "a half-submerged gasometer, with the skeleton of an ironfoundry erected on its sullen dome." The book is written in a patriotic spirit all the way through, and while he is describing the beauties and charms of foreign lands on the other side of the lantern, he is not unmindful of home, but constantly turns homeward, like a patriot, with a kindly and genial comparison.

THE PUERPERIUM, OR THE MANAGEMENT OF THE LYING-IN WOMAN AND NEWBORN INFANT. By C. NEPEAN LONGRIDGE, M.D., Ch.B.(Vict.), F.R.C.S.(Eng.), M.R.C.P. (Lond.), Pathologist and Registrar, late Resident Medical Officer, at Queen Charlotte's Lying-in Hospital. (London: Adlard and Son.)

BOOKS of this description are produced in such rapid succession that one must necessarily conclude that they contain precious information for which the demand is large and sustained. Dr. Longridge has produced a work which will prove of great service to the student-practitioner just emerging from the classical realms of studentship into the more plebeian and humdrum work of general practice. Many of his idols will be shattered here. His faith in himself and in human nature will become tremulous. In such periods of emotion he will find great comfort from the thoroughly clear and practical and kindly advice placed within his reach in the volume before us. The care and skill which a practitioner shows in guiding his patients through the puerperal period will probably be the keystone of his future success, and it is by attention to matters of detail, brought into prominence and impressed upon his readers by the author of this important work, that prosperity can be achieved.

THE EARLY LIFE OF SIR THOMAS BROWNE.

THE recently issued volume of the Transactions of the Norfolk and Norwich Archæological Society (Vol. XVI., 1906, pp. 132-146) contains an interesting contribution by Mr. Charles Williams, F.R.C.S.E., relating to Sir Thomas Browne, author of "An Inquiry into Vulgar Errors," "Religio Medici," and "Urn Burial." Mr. Williams prints the will of Thomas Browne, father of the great Norwich physician, and reproduces a curious portrait group of the Browne family, the original of which is in Devonshire House, Piccadilly. The group shows father, mother, and three daughters, with Thomas, about the age of three, sitting on his mother's knee nursing a black rabbit. Thomas Browne the elder was himself the son of Thomas of Upton, near Chester, and of his wife Elizabeth, daughter of Henry Birkenhead, of Huxley, Cheshire. The father of the physician was the third son, and carried on business as a mercer in the parish of St. Michael-le-Querne, Cornhill. He married Anne, the daughter of Paul Garraway, of Lewes, by whom he had three daughters, of whom nothing more is known, and the one son, Thomas. It is curious that Sir Thomas, in that part of his correspondence which has been hitherto published, makes no allusion to any member of his family, but as many letters still remain unpublished in the Rawlinson and Sloane collections at the British Museum, some information may still be forthcoming in addition to the interesting facts which Mr. Charles Williams has been so successful in obtaining. Thomas Browne was eight years old when his father died in 1613, and he entered Winchester College on August 20, 1616, in his eleventh year, his name standing sixth on the roll of scholars. He stayed at Winchester for seven years, till his scholarship lapsed on the completion of his eighteenth year, and he probably spent his holidays with his mother, who continued to live in Cheapside. It is necessary to emphasise this, as an ill-natured story has found credence that Anne Browne, after the death of her first husband, repudiated her children—turned them out of doors "helpless and unprotected"—on her marriage with Sir Thomas Dutton, of Isleworth. On leaving Winchester Thomas Browne entered as a Fellow Commoner at Broadgates Hall, Oxford, matriculating December 5, 1623, the year before the Hall was incorporated as Pembroke College. On the occasion of the conversion of Broadgates Hall to Pembroke College on August 5, 1625, Browne, as the Senior Fellow Commoner, was called upon to deliver the first of the three Latin orations. The oration has been preserved, and Mr. Charles Williams has published an excellent translation of it into English, by Professor E. von B. Bensly. At the end of six years of college life Browne took his Master of Arts degree on June 11, 1629, and after travelling for four years in France, Spain, Holland, and Italy, where he took the degree of Doctor of Medicine, he settled at Shibden Dale, a remote spot on the hillside three miles from Halifax, to recruit from the perils and fatigues through which he had passed. In this tranquil retreat he wrote his famous tract, "Religio Medici," and having stayed there during the years 1634 and 1635, he finally moved to Norwich in 1636, where he practised medicine with great success for forty-six years. All who love Sir Thomas Browne and his works—and they increase in number yearly—will feel under a debt of gratitude to Mr. Charles Williams for the present paper. It clears up for ever the imputations which have too long rested on Anne Browne. The author shows how these arose, and that they obtained currency by the carelessness, to use no stronger word, of no less a person than Dr. Samuel Johnson, who prefixed a life of the author to his edition of the "Christian Morals" published in 1756.

